# Supplementary material for: The catecholamine precursor Tyrosine reduces autonomic arousal and decreases decision thresholds in reinforcement learning and temporal discounting
Source: PLoS Comput Biol. 2022 Dec 22;18(12):e1010785. doi: 10.1371/journal.pcbi.1010785 (PMC9822114; doi:10.1371/journal.pcbi.1010785)
Supplement: S1 Text — (DOCX) [file pcbi.1010785.s001.docx]

**Supporting Information**

**Results**

*Posterior predictive checks*

Posterior predictive checks (see ’Methods’ section for details) showed that the DDM_s_ reproduced the effect of decision conflict on participants’ RTs and choice patterns best in comparison to alternative model formulations for both the seq. RL task (Fig S1) and for the temporal discounting data (Fig S2).

*Sequential RL task, drift diffusion model*

Fig S3 depicts the posterior distributions of all group-level mean parameter from the DDM_s_ for the seq. reinforcement learning data following placebo intake (left column, light grey plots) and their respective shifts related to tyrosine intake (right column, dark grey plots). Boxplots depict 80% and 90% HDIs.

*Sequential RL task, softmax model*

We also implemented an extension of an established RL model [1,2] that is based on softmax action selection instead of a DDM implementation (see Methods section). This model included separate parameters for S1 and S2 learning rates, a separate decay rate for unchosen options in both stages, model-free and model-based $\beta$ weights for S1 and a$\beta$ weight for S2 *Q*-value differences. Note that this model formulation includes the nested versions with one learning rate, no decay rate, no perseveration, no model-based and or no model-free effects as special cases, where the respective posteriors are centered at 0. Kruschke (2011) [3] suggests to examine the posterior distributions of the full model in such cases, rather than performing a model comparison across all nested versions. Similar to the mixed regression effects of reward and the reward*transition interaction above, we observed substantial contributions of both model-free and model-based values to S1 choice probabilities ($\beta_{MF}$,$\beta_{MB}$ in Fig S4I and S4J). Participants also exhibited choice preservation in S1 and their choices in S2 were affected by S2 Q-value differences ($\rho$,$\beta_{S2}$ in Fig S4D and S4K). Learning rates for updating of S1 and S2 model-free Q-values significantly differed (90% highest density interval (HDI) of $\eta_{S1}$ and $\eta_{S2}$did not overlap, Fig S4A and S4B) and unchosen choice option Q-values substantially decayed towards the mean ($\eta_{decay}$, Fig S4C).

Tyrosine led to a significant decrease in the decay rate $\eta_{decay}$ in the softmax model (mean[90% HDI] = -.27 [-.48, -.08], directional Bayes Factor (dBF) = .01, Fig S4G). According to dBF analysis, participants exhibited a substantial increase in updating of S1 model-free Q-values through heightened learning rates $\eta_{S1}$(mean[90% HDI]=.54[-.07,1.09], dBF=16.3; Fig S4E). All other posterior distributions of tyrosine-related parameter changes showed a substantial overlap with zero (80% HDIs, Fig S4).

*Temporal discounting task, softmax model*

We also modeled choice data in the temporal discounting task using a hyperbolic discounting model with standard softmax-action selection (see methods section). In this model, we found no evidence for a modulatory effect of tyrosine supplementation on discount rate log(k) (mean [90% HDI]: $k_{s}$=-.7 [-.28, .17], Fig S5). We also found no evidence for a modulatory effect of tyrosine on modeled choice stochasticity (mean [90% HDI]: $\beta_{s}$=.01 [-.04, .07], Fig S5).

*Correspondence between task performance and physiology*

In an exploratory attempt, we tested for associations between task performance under placebo and physiological arousal measures (spontaneous eye blink rate, pupil dilation, pupil dilation variability, heart rate, heart rate variability; note that these values were averaged across placebo and tyrosine t0 baseline measurements), as well as tyrosine-related changes of these. We restricted these exploratory analyses to core aspects of seq. RL and temporal discounting, and related evidence accumulation processes to reduce multiple testing burden. For the seq. RL task, we focused on potential links between physiological measures and average payout per trial as an agnostic measure of general task performance, the impact of model-free and model-based Q-values on evidence accumulation speed ($\beta_{mf}$, $\beta_{mb}$), as well as participants’ average (across task stages) decision thresholds $\alpha=mean(\alpha_{1},\alpha_{2})$ and non-decision times $\tau=mean(\tau_{1},\tau_{2})$. In addition, we tested for associations between tyrosine related changes in pupil dilation variability and heart rate (spontaneous eye blink rate, pupil dilation and heart rate variability were unaffected by tyrosine), and tyrosine associated shifts in decision thresholds $s_{\alpha}=mean(s_{\alpha_{1}}, s_{\alpha_{2}})$and model-based control $s_{\beta_{mb}}$. Baseline pupil dilation was negatively associated with individual non-decision times during seq. RL ($\tau$; r=-.5, p=.007; Fig S6A) and pupil dilation variability was positively correlated with the impact of model-free Q-values on trial-wise drift-rates ($\beta_{mf}$; r=.49, p=.009; Fig S6B). All other tested associations were non-significant (all p>.14).

For testing potential associations between temporal discounting performance under placebo and physiological arousal measures, we focused on % LL choices as a model agnostic measure, discounting parameter log($k$), bias towards LL/SS choices $z$, decision thresholds$\alpha$, non-decision time $\tau$ and evidence accumulation speed $\nu$. In addition, we tested for associations between tyrosine related changes in pupil dilation variability and heart rate, and tyrosine associated shifts in discounting$s_{log(k)}$ and decision thresholds $s_{\alpha}.$We found that higher pupil dilation at baseline was associated with more impatient (fewer LL) choices during temporal discounting (r=-.55, p=.002 ; Fig 7A - main manuscript). In line, pupil dilation at baseline predicted steeper discounting (log($k$); r=.51, p=.005; Fig S6C) and higher pupil dilation was associated with a significant bias towards impatient SS choices ($z$; r=-.63, p=3.0*10^-4; Fig 7B – main manuscript). Higher pupil dilation variability at baseline was related to longer non-decision times ($\tau$; r=.46, p=.01). In addition, higher spontaneous eye blink rate at baseline was associated with lower decision thresholds during temporal discounting ($\alpha$; r=-.4, p=.03). With respect to tyrosine related changes in physiological arousal, greater pre-post heart rate changes following tyrosine compared with placebo were associated with tyrosine related shifts in temporal discounting ($s_{log(k)}$; r=.61, p=5.76*10^-4; Fig 7C – main manuscript). All other tested associations were non-significant (all p>.06).

However, when adjusting for False Discovery Rate [4] only the associations between pupil dilation and % LL choices, as well as bias towards SS choices, and between tyrosine related modulation of pre-post heart rate changes and tyrosine related shifts in temporal discounting remained significant (all other p-values >=.005; FDR adjusted p-value = .003).

**References**

1. Daw ND, Gershman SJ, Seymour B, Dayan P, Dolan RJ. Model-based influences on humans’ choices and striatal prediction errors. Neuron. 2011;69: 1204–1215. doi:10.1016/j.neuron.2011.02.027

2. Otto AR, Raio CM, Chiang A, Phelps EA, Daw ND. Working-memory capacity protects model-based learning from stress. Proc Natl Acad Sci U S A. 2013;110: 20941–20946. doi:10.1073/pnas.1312011110

3. Kruschke JK. Bayesian Assessment of Null Values Via Parameter Estimation and Model Comparison. Perspect Psychol Sci. 2011;6: 299–312. doi:10.1177/1745691611406925

4. Benjamini Y, Hochberg Y. Controlling the False Discovery Rate: A Practical and Powerful Approach to Multiple Testing. Journal of the Royal Statistical Society Series B (Methodological). 1995;57: 289–300.
