# Supplementary figures and images for: The catecholamine precursor Tyrosine reduces autonomic arousal and decreases decision thresholds in reinforcement learning and temporal discounting
Source: PLoS Comput Biol. 2022 Dec 22;18(12):e1010785. doi: 10.1371/journal.pcbi.1010785 (PMC9822114; doi:10.1371/journal.pcbi.1010785)

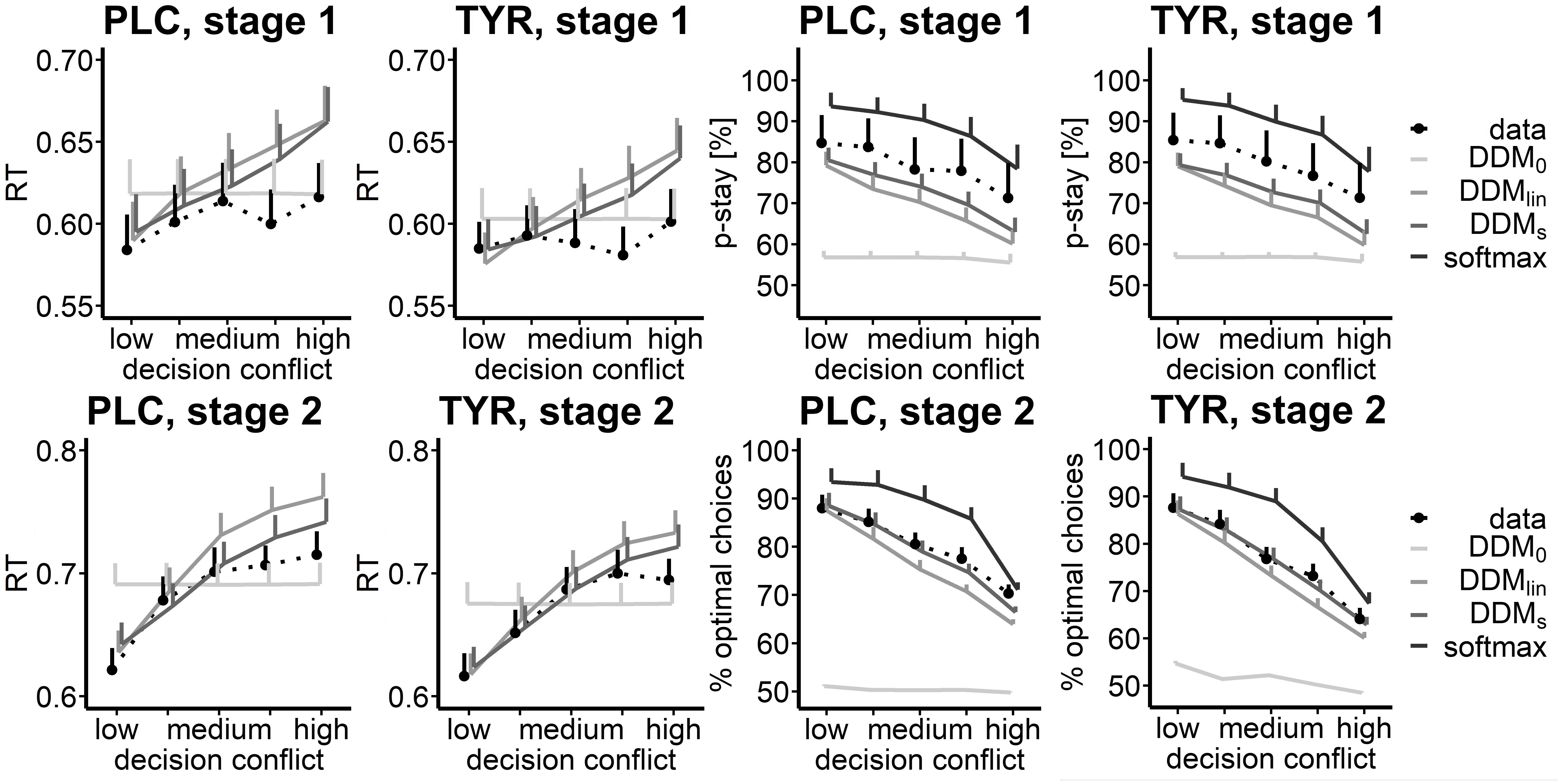

Supplement: S1 Fig — In each plot the dotted line depicts participants’ median RTs or mean choice behavior. Solid lines depict the median RTs and mean choice behavior drawn from 500 simulations of each of the different DDM formulations, as well as of a standard softmax model for choice data. The upper row depicts RT data and simulations and participants’ probability to choose the same action as in the previous trial for the first decision stage S1 in relation to the options value differences (‘decision conflict’). The lower row depicts S2 RT data and fraction of optimal choices in S2 (highest value option chosen) of participants and related model simulations. (JPG) [file pcbi.1010785.s002.jpg]

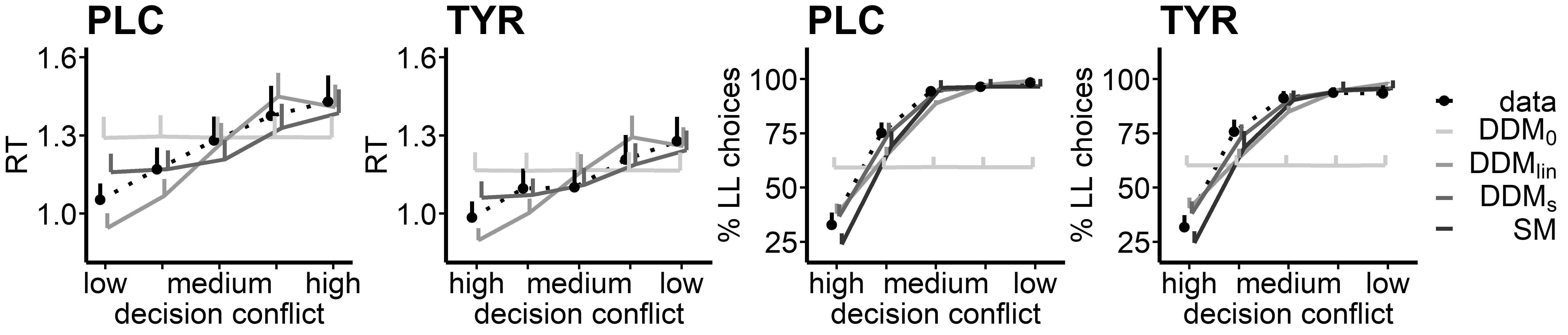

Supplement: S2 Fig — In each plot the dotted line depicts participants’ median RTs or mean % LL choices. Solid lines depict the median RTs or mean LL choices drawn from 500 simulations of each of the different DDM formulations, as well as of a standard softmax model for choice data. Data and simulations are plotted in relation to the absolute difference of LL (subjective values) and SS choice options. (JPG) [file pcbi.1010785.s003.jpg]

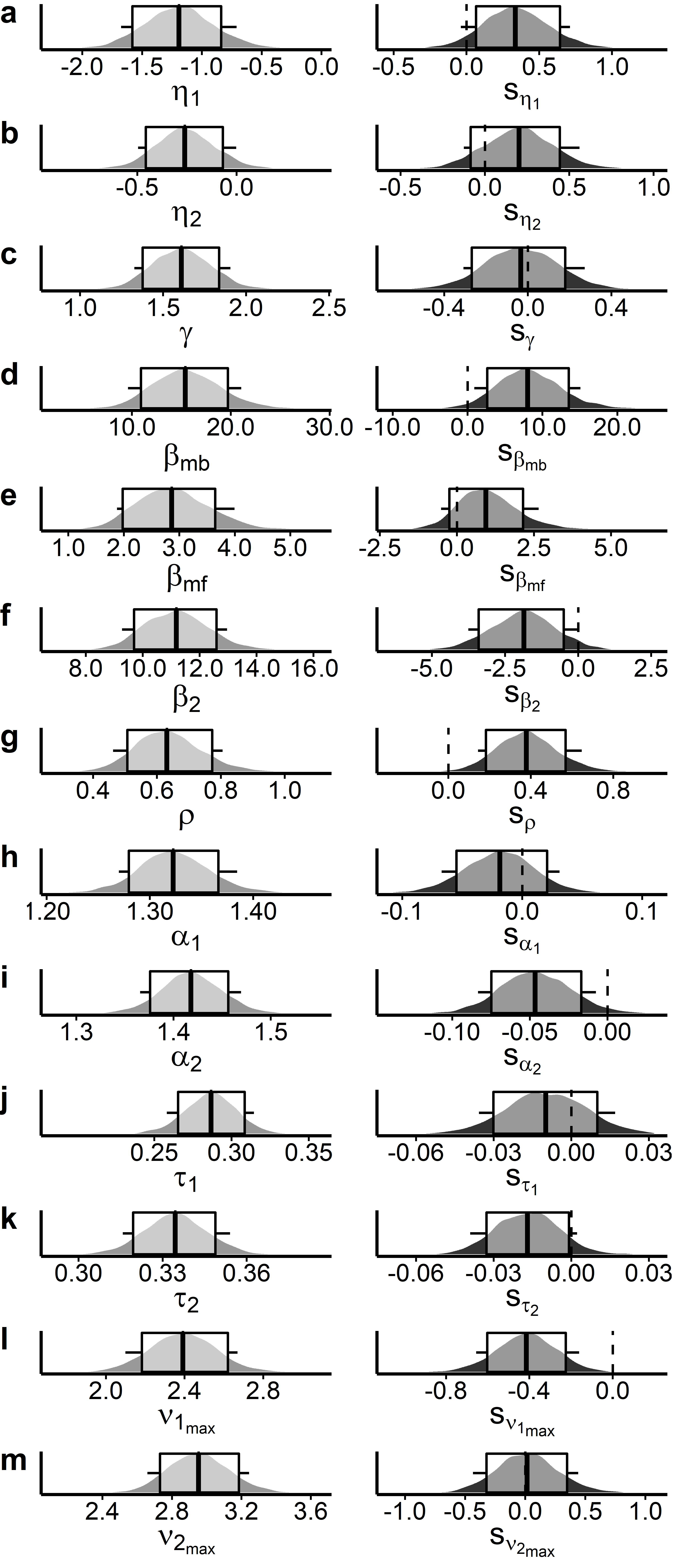

Supplement: S3 Fig — Posterior distributions of all group-level mean parameter from the DDMs for the seq. reinforcement learning data following placebo intake (left column, light grey plots) and their respective shifts related to tyrosine intake (right column, dark grey plots). Boxplots depict 80% and 90% HDIs. Depicted parameters are (A)-(C) learning rate η1 and η2 for prediction error related updating in S1and S2, and value decay rate of unchosen options γ, (D)-(F model-based parameter βmb, model-free parameter βmf and drift-rate modulation by value differences in S2, (g) perseveration parameter ρ, (h)-(i) decision thresholds in stage S1 and S2 α1, α2, (j)-(k) non-decision times in S1 and S2 τ1, τ2, and (l)-(m) S1 and S2 drift-rate asymptotes ν1max,ν2max. (JPG) [file pcbi.1010785.s004.jpg]

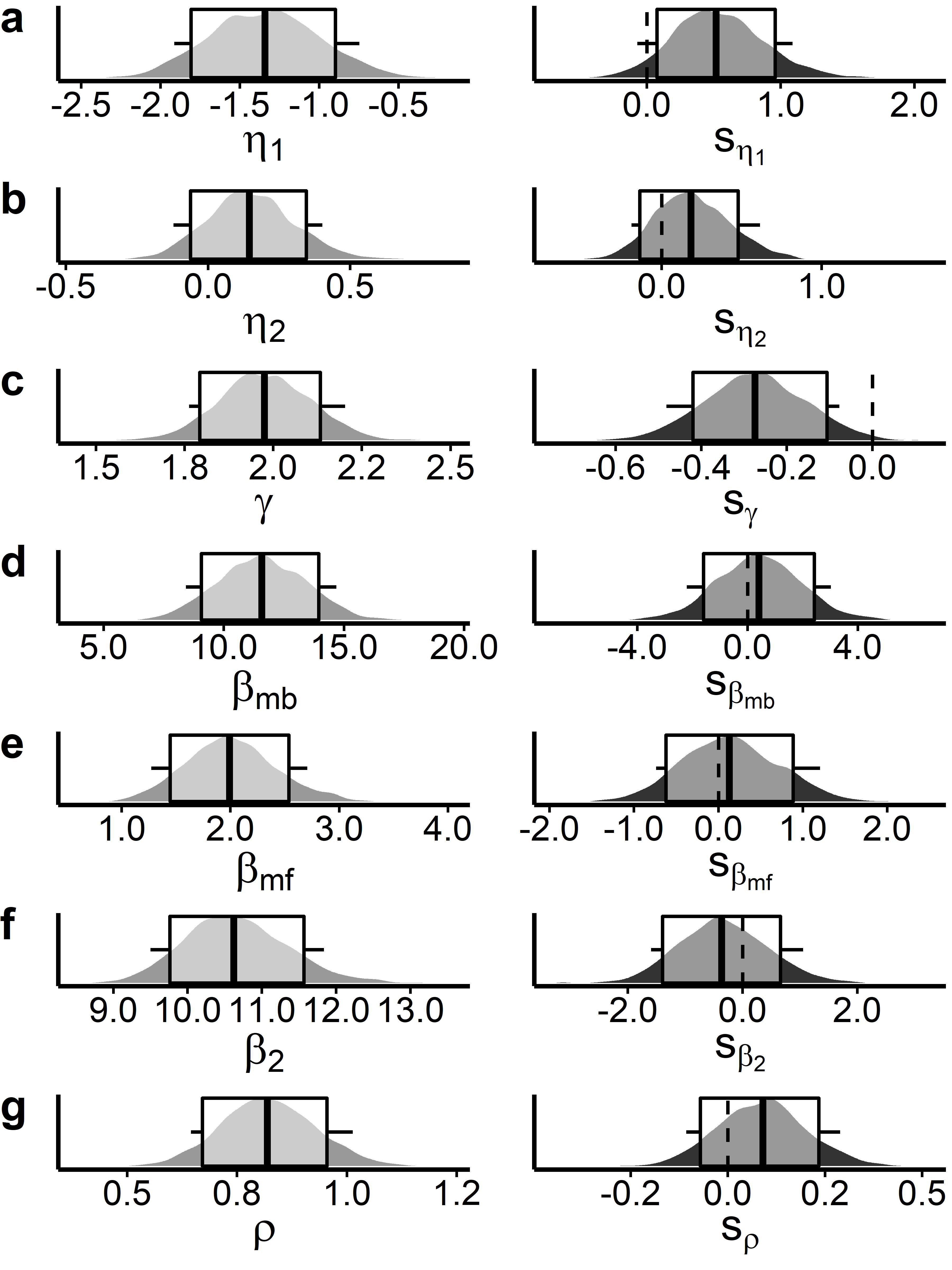

Supplement: S4 Fig — Posterior distributions of the group-level means of the softmax model of the seq. RL task data following placebo intake (left column, light grey plots) and their respective shifts related to tyrosine intake (right column, dark grey plots). Boxplots depict 80% and 90% HDIs. Depicted parameters are from left to right: (A) learning-rate in S1 η1, (B) learning-rate in S2 η2, (C) decay rate of unchosen options γ, (D) model-based βmb weight and (E)(E) model-free βmf weight, (F S2 stage Q-value β2 weight and (g) choice perseveration ρ. (JPG) [file pcbi.1010785.s005.jpg]

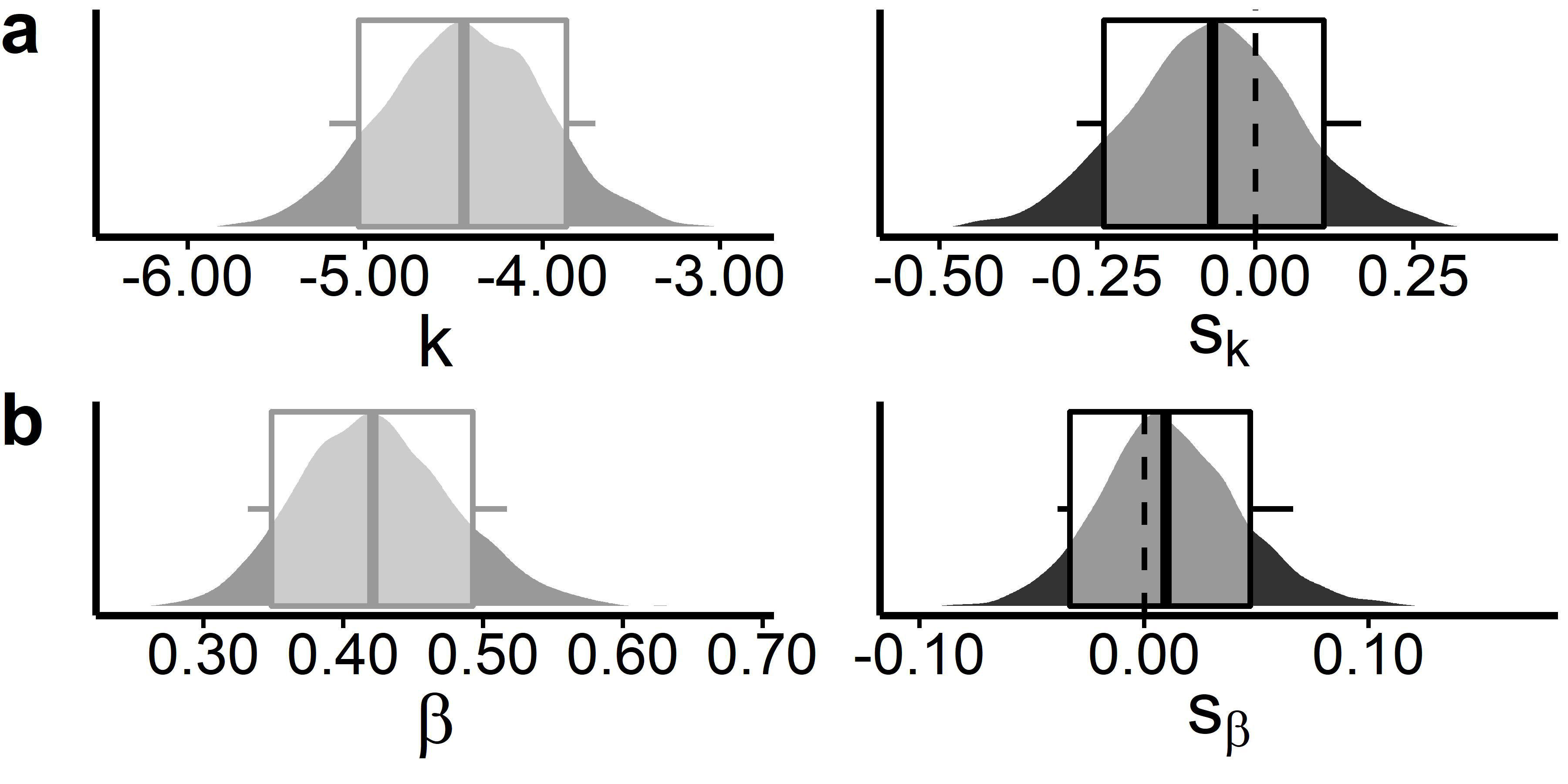

Supplement: S5 Fig — Posterior distributions of the group-level means of the softmax model of the temporal discounting task data following placebo intake (left column, light grey plots) and their respective shifts related to tyrosine intake (right column, dark grey plots). Boxplots depict 80% and 90% HDIs. Depicted parameters are: (A) Discount rate log (k) and (B) softmax inverse temperature β. (JPG) [file pcbi.1010785.s006.jpg]

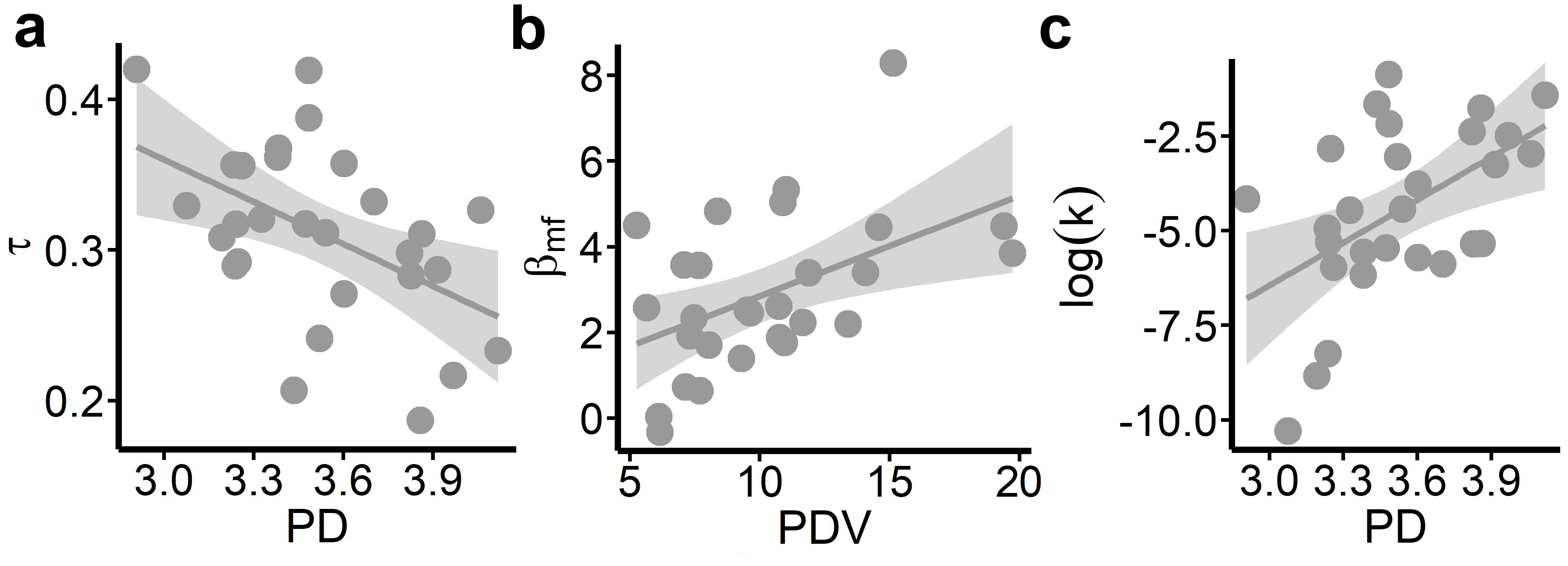

Supplement: S6 Fig — (A) Participants’ pupil dilation at baseline (mean of t0 physiological measurements across tyrosine and placebo) was predictive of participants’ average (S1 & S2) non-decision times (τ) under placebo during seq (r = -.5, p = .007). RL. (B) Individual pupil dilation variability (PDV) at baseline was associated with the degree of drift-rate modulation by model-free Q-values (βmf) during seq. RL under placebo (r = .49, p = .009). (C) Pupil dilation at baseline was also related to temporal discounting log(k) (r = .51, p = .005). Note, that the depicted associations fell short of significance after adjusting for False Discovery Rate (all p-values > FDR adjusted p-value = .003). (JPG) [file pcbi.1010785.s007.jpg]
