# Supplementary material for: The catecholamine precursor Tyrosine reduces autonomic arousal and decreases decision thresholds in reinforcement learning and temporal discounting
Source: PLoS Comput Biol. 2022 Dec 22;18(12):e1010785. doi: 10.1371/journal.pcbi.1010785 (PMC9822114; doi:10.1371/journal.pcbi.1010785)
Supplement: S1 Table — SE = standard error; YOE = years of education; BDI-II: Beck Depression Inventory-II; BIS-15: Barratt-Impulsiveness Scale (15 items); BIS/BAS: Behavioral Inhibition and Behavioral Activation System. (DOCX) [file pcbi.1010785.s008.docx]

|  | mean (range) | se |
| --- | --- | --- |
| age | 25.25 (20-34) | 0.75 |
| BMI | 24.13 (19.6-28.89) | 0.48 |
| YOE | 12.5 (11-15) | 0.15 |
| income | 1023.93 (0-5000) | 169.7 |
| BDI-II | 5.11 (0-18) | 0.8 |
| BIS-15 | 34.18 (26-44) | 1.05 |
| BIS(BIS/BAS) | 2.35 (1.57-3.29) | 0.08 |
| BAS(BIS/BAS | 3.05 (2.46-3.85) | 0.06 |

**Table S1**. Study sample characteristics (N=28). SE= standard error; YOE=years of education; BDI-II: Beck Depression Inventory-II; BIS-15: Barratt-Impulsiveness Scale (15 items); BIS/BAS: Behavioral Inhibition and Behavioral Activation System.
