# Supplementary material for: The catecholamine precursor Tyrosine reduces autonomic arousal and decreases decision thresholds in reinforcement learning and temporal discounting
Source: PLoS Comput Biol. 2022 Dec 22;18(12):e1010785. doi: 10.1371/journal.pcbi.1010785 (PMC9822114; doi:10.1371/journal.pcbi.1010785)
Supplement: S2 Table — (DOCX) [file pcbi.1010785.s009.docx]

| S1 - RTs | $\beta$ | SE | t | p |
| --- | --- | --- | --- | --- |
| interc | .6 | 0.02 | 34.12 | **<2*10^-16** |
| rew | 8.55*10^-4 | 2.26*10^-3 | .38 | .71 |
| **trans** | **5.14*10^-3** | **1.29*10^-3** | **2.73** | **7.41*10^-3** |
| **TYR** | **-8.9*10^-3** | **1.86*10^-3** | **-4.8** | **1.61*10^-6** |
| rew*trans | 1.88*10^-3 | 1.88*10^-3 | -1.0 | .32 |
| rew*TYR | -4.43*10^-4 | 1.85*10^-3 | -.24 | .81 |
| trans*TYR | -1-53*10^-3 | 1.84*10^-3 | -.83 | .41 |
| rew*trans*TYR | -2.77*10^-3 | 1.85*10^-3 | -1.5 | .13 |

**Table S2.** Effects on participants S1 RTs from a mixed effects regression analysis (rew=reward; trans=state transition; TYR=tyrosine).
