# Supplementary material for: The catecholamine precursor Tyrosine reduces autonomic arousal and decreases decision thresholds in reinforcement learning and temporal discounting
Source: PLoS Comput Biol. 2022 Dec 22;18(12):e1010785. doi: 10.1371/journal.pcbi.1010785 (PMC9822114; doi:10.1371/journal.pcbi.1010785)
Supplement: S3 Table — (DOCX) [file pcbi.1010785.s010.docx]

*Model-comparison, predictive accuracy*

Depicted below is the model fit comparison according to Watanabe-Akaike Information Criterion (WAIC) and the estimated log pointwise predictive density (elpd).

|  | drift-rate modulation | | WAIC | -elpd | -$\Delta$elpd | 95% CI (-$\Delta$elpd) |
| --- | --- | --- | --- | --- | --- | --- |
| Temp. Discount. |  |  | |  |  |  |
| **DDM_0_** | - | 10946 | | 5518 | 2027 | 1755 - 2298 |
| **DDM_lin_** | linear | 8176 | | 4141 | 650 | 513 - 788 |
| **DDM_s_** | sigmoid | 6880 | | 3491 | - | - |
| *Seq. RL* |  |  | |  |  |  |
| **DDM_0_** | - | 20989 | | 10548 | 8932 | 7997 - 9868 |
| **DDM_lin_** | linear | 3943 | | 2021 | 405 | 244 - 567 |
| **DDM_s_** | sigmoid | 3136 | | 1616 | - | - |

**Table S3.** Model fit comparison of the DDMs in the temporal discounting & the seq. RL task via the Watanabe-Akaike Information Criterion (WAIC), the estimated log pointwise predictive density (elpd), and its difference to the winning model (DDM_s_).
