# Supplementary material for: The catecholamine precursor Tyrosine reduces autonomic arousal and decreases decision thresholds in reinforcement learning and temporal discounting
Source: PLoS Comput Biol. 2022 Dec 22;18(12):e1010785. doi: 10.1371/journal.pcbi.1010785 (PMC9822114; doi:10.1371/journal.pcbi.1010785)
Supplement: S4 Table — RL choice data, respectively. Values are computed via simulations based on 500 samples drawn from each of the respective single subject parameters’ posterior distributions. (DOCX) [file pcbi.1010785.s011.docx]

|  | softmax | DDM_0_ | DDM_lin_ | DDM_s_ |
| --- | --- | --- | --- | --- |
| **TD task** | 87 (66-93) | 64 (49-100) | 76 (60-87) | 83 (64-96) |
| **seq. RL, S1** | 80 (50-96) | 56 (49-77) | 73 (51-92) | 75 (51-95) |
| **seq. RL, S2** | 81 (55-94) | 51 (47-57) | 72 (51-87) | 75 (52-90) |

**Table S4**. Proportions of correctly predicted binary choices (mean (range)) for the temporal discounting (TD) task data and for both stages (S1, S2) of the seq. RL choice data, respectively. Values are computed via simulations based on 500 samples drawn from each of the respective single subject parameters’ posterior distributions.
