# Supplementary material for: The catecholamine precursor Tyrosine reduces autonomic arousal and decreases decision thresholds in reinforcement learning and temporal discounting
Source: PLoS Comput Biol. 2022 Dec 22;18(12):e1010785. doi: 10.1371/journal.pcbi.1010785 (PMC9822114; doi:10.1371/journal.pcbi.1010785)
Supplement: Attachment — Submitted filename: 2022-08-04_Review_PCOMPBIOL-D-22-00800.docx [file pcbi.1010785.s012.docx]

**PCOMPBIOL-D-22-00800**

In their study "The catecholamine precursor Tyrosine reduces autonomic arousal and decreases

decision thresholds in reinforcement learning and temporal discounting" David Mathar and colleagues investigate the impact of a catecholaminergic agent on value-based decision making. Using a double-blind, placebo-controlled, within-subject design in combination with computational modelling the authors show that a single-dose of L-Tyrosine alters psychophysiological parameters in a goal-directed manner.

This is an interesting study that highlights the promising nature of single-dose application of Tyrosine to study cognition. In order to make claims about the impact of their pharmacological manipulation the authors employed a thoughtful and solid task design as well as state-of-the-art methodology. Most interpretations and conclusion drawn from the results seem justified. The manuscript is well-written and well-motivated. However, I also have some minor concerns on the current manuscript which I would like to ask the authors to clarify and reply.

MINOR COMMENTS

(1) Most importantly, I was surprised to read that the choice for the reinforcement learning model is not fully in line with previous realizations in the literature. While most modelling choices follow standard procedures, the stage1 choice rule (equation 8) deviates from previous instances of similar reinforcement learning models. To my knowledge, the conventional procedure to convert (and combine) model-free and model-based action values into action probabilities is (1) to derive net action values based on a model-basedness parameter and then (2) submit those net action values to the softmax decision rule. This is how it is reported in both early work (e.g. Gläscher et al. 2010) as well as a current DDM RL framework (Shahar et al., 2019). In contrast, the authors take a different approach. They refrain from calculating net action values based on a model-basedness parameter but rather introduce separate temperature parameters for model-free and model-based action vales within their softmax decision rule. Although I acknowledge that the softmax model is only used for a comparison purpose to the DDM models, a justification for the deviation from standard model formulations is missing in the current manuscript. Arguably, the deviation in model specification introduced by the authors has a significant impact on interpretability of the model. Moreover, it is not fully clear to me how the different parameters for the authors' model (separate model-free and model-based temperature) relate to the standard model (model-basedness and temperature). I would be interesting to learn if the two different model instances are recoverable.

(2) I find the conclusion that model-based RL was improved by tyrosine (line 864) to be misleading. DDM shows that the beta parameter significantly improves for tyrosine. However, this scaling parameter (similar to the temperature parameter in the softmax model) is not involved in learning per se but only in decision making, i.e. translating learned values into action. Given the current form of the computational model, no statements about (the strength of) learning in a reinforcement learning framework can be made. For such a claim, a different model (e.g. model-free and model-based learning rates) or more event-related measures (e.g. EEG, fMRI or pupillometry) seem necessary.

(3) Throughout the manuscript, the visualization could be improved. Personally, I believe visualization to be a key factor for promoting understanding of complex finding. As a positive example I have to highlight the use of violin plots which allow to depict information in an intuitive manner across both the individual as well as group level.

(3.1) The manuscript could profit from a figure that illustrates the design and structure of the tasks. Especially the sequential RL task is quite complicated. Understanding for readers who are not familiar with the task (or its modifications based on Kool et al.) would be fostered.

(3.2) On a similar note. In order to visualize the model-free and model-based contributions as revealed by the model-agnostic analysis, I suggest that the authors add a figure with both hypothetical (i.e. pure model-free and model-based) as well as empirical stay probabilities per condition.

(3.3) In order to highlight plot differences within and between figures, I prefer to use captions.

(4) Contrary to my expectations, administration of L-Tyrosine seemingly leads to a decrease in physiological arousal, as indicated by pupil dilation and heart rate. Although the authors point to relevant literature on possible hypotensive effects of Tyrosine in stressful situation, the counterintuitive nature of the findings seems to be underrepresented in the current manuscript. Increased dopamine and noradrenaline plasma levels are reportedly associated with an overall (if mild) increase in physiological arousal.

(5) Contrary to the data and code availability statement, modeling code is not available on OSF.

FURTHER COMMENTS

- Why are the variables LL magnitude and delay median-split before submission to the generalized mixed effects regression of the model-agnostic analysis of the temporal discounting task? Admittedly, I am not an expert for analyses of this task, but a median split seems to be overly simplistic, given the continuous nature of the two variables (16 and 8 steps respectively).
- Although the methodology for the parameter comparison of the temporal discounting DDM is sound, the reported results need further clarification, as the sole strong effect (decision threshold, see fig 6f) is not obvious in the main text (line 627-631).
- Line 528: Figure 1b should read 2b
