# Supplementary material for: The catecholamine precursor Tyrosine reduces autonomic arousal and decreases decision thresholds in reinforcement learning and temporal discounting
Source: PLoS Comput Biol. 2022 Dec 22;18(12):e1010785. doi: 10.1371/journal.pcbi.1010785 (PMC9822114; doi:10.1371/journal.pcbi.1010785)
Supplement: Attachment — Submitted filename: Point_by_point_letter.docx [file pcbi.1010785.s013.docx]

**Point by point response to the reviewers’ concerns**

Reviewer #1

(1) In short, from the introduction it is not clear to me _why_ the authors decided to run this particular study. Of course, I understand that L-Tyrosine results in increases of dopaminergic availability, but given the previous literature, which found mixed findings regarding dopamine and the forms of decision making under investigation, it is not clear how this particular study will address this uncertainty. Is there a reason to believe that this form of supplementation more reliably increases dopamine? Is the double-blind placebo-controlled design particularly new? This is not clear.

As it stands now, the paper simply discusses two, relatively unrelated, forms of decision making sequentially, and then motivates the study through prior uncertainty. Aren’t there more theoretically interesting reasons to have run the study? What are some proposed theoretical roles for dopamine in model-based RL and temporal discounting? How would these results address these questions?

Moreover, I think the paper overpromises a combination of two tasks (line 100), but (as far as I understand) there is relatively little interaction between the tasks and the datasets they produce. The models are fit separately, and their results are interpreted separately. It’s unclear why the authors chose these particular tasks, and what they think they are getting by running them both in participants in the same session. Of course, efficiency and convenience are good reasons for doing this, but from the perspective of the paper it does not come across as particularly strong.

The discussion does not help clear this up. Analogous to the introduction, the results are discussed piece by piece, without drawing much attention to potential mutual mechanisms. The notable exception here is the discussion of RT and DDM threshold effects. However, most of the discussion reads as if the authors conduct two parallel studies that needed to be discussed in tandem. I also missed some more speculative theoretical discussion on the mechanisms underlying these L-tyrosine effects.

I think the authors should either considering making clearer what the benefit of the current setup is over prior studies (especially focusing on the use of these two tasks side-by-side). Alternatively, they can consider breaking up this one paper in two, so that they can pay more attention to the interesting but relatively separate literatures underlying these tasks, without having to attempt to unite them under one banner.

*We apologize for not formulating the motivation for this study more precisely. First of all, L-tyrosine is highly interesting as it can be freely retrieved as a nutritional supplement making it easily accessible. For this reason, it is discussed in the context of potentially beneficial “lifestyle” interventions (Kita et al., 2018; Kühn et al., 2019). To date, there exist several studies that investigated effects of L-Tyrosine supplementation on cognitive performance. But there are several short comings here: Most studies investigated effects of tyrosine on working memory capacity (e.g. Thomas et al., 1999; Colzato et al., 2013) or more simple perceptual-motor tasks (e.g. Deijen et al., 1999) with many studies testing beneficial effects of tyrosine in these tasks under some sort of stress. However, reinforcement learning and value-based decision-making are known to be strongly modulated by catecholamine transmission and thus are potential targets of tyrosine supplementation. Such constructs might arguably have an even higher relevance to everyday decision-making due to their consistent link with maladaptive behaviors and trans-diagnostic relevance across (sub-) clinical psychiatric symptom dimensions. More specifically, both model-based reinforcement learning and temporal discounting performance have emerged as two candidates delivering on the promise of the Research Domain Criteria as trans-diagnostic characteristics that seem to go awry in a broad range of psychiatric illnesses. Thus, if performance in these two tasks is modulated by tyrosine supplementation, this has potential clinical and applied implications. In addition, existing supplementation studies lack comprehensive model-based approaches that are regularly applied in the context of pharmacological studies. Therefore, a second goal was to more precisely characterize potential cognitive effects of tyrosine supplementation on specific computational processes.*

*Notably, despite the separate modeling of the two task data sets there is an interesting overlap in DDM model parameters such as decision thresholds and related shifts following tyrosine. In the discussion section ‘Tyrosine reduced decision thresholds’ of our manuscript, we elaborate on this consistent effect in both tasks.*

*We now reframed the introduction section to emphasize our motivation for including both tasks in this study.*

(2) The authors claim that they changed the two-step task so that model-based RL yields increased reward rate. However, they do not directly demonstrate this. There is suggestive evidence, namely that reward rate is correlated with differences in RT between rare and common trials, but this is a distant proxy for model-based control. Why did they not demonstrate the advantage of model-based RL with simulations? Why did they not correlate decision making variables (such as the temperature parameter or the interaction effect on stay probabilities) with reward rate?

*Thanks for the opportunity to clarify this point. Differences in response times following rare vs. common transitions are one of the key signatures of model-based control during reinforcement learning in this task (see Otto et al., 2015; Shahar et al., 2019). However, if we run the analysis suggested by this Reviewer, we indeed observe similar correlations with reward rate and individual model-based beta parameter (+ shift following tyrosine) of the DDM_s_ (PLC: r=0.67, p=1.06*10^-4; TYR: r=0.39, p=.04), as well as with the beta weights of the individual interaction effects of reward*transition from the mixed effects regression model (PLC: r=* *0.74, p=6.08*10^-6; TYR: r=* *0.72, p=1.41*10^-5).*

*We focused on differences in S2 response times as a marker of model-based control, as this depicts a ‘raw’ behavioral measure without any model assumptions/constraints compared to interaction terms from a mixed effects regression model or means of DDM parameter distributions. We now also included the correlations between reward*transition interaction and model-based beta parameter with mean payoffs in the revised manuscript (lines 571-73 & 595-98).*

(3) For the modeling of the two-step task, I urge the authors to also model response key perseveration in addition to the stimulus perseveration (see Bolenz et al., 2019, eLife for an example).

*Thanks for this idea. We now also tested a DDM_s_ implementation that includes response key perseveration in addition to stimulus perseveration in S1. However, model comparison revealed a marginally poorer fit of this model compared to the DDM_s_ without response key perseveration (WAIC(DDM_s-key_)=3136.4 vs. WAIC(DDM_s_)=3136). With this limitation in mind, we nonetheless examined the posterior distributions of this model. This revealed no evidence of response key perseveration under placebo (90% HDI centered around zero). There was a negative shift in response key perseveration following tyrosine, indicating higher switching between response keys. All other parameter distributions remained similar. Due to the inferior model fit, we did not include this model into our revised manuscript. Below we attached the posterior distribution of the respective group-level parameter following placebo and its respective shift following tyrosine supplementation.*

Posterior parameter distributions of the (group-level) response key perseveration parameter of the DDM_s-key_ following neutral cue-exposure (left column, grey plots) and its respective shift as change related to tyrosine vs placebo supplementation (right column, black plots). Boxplots depict 80% and 90% HDIs.

(4) It was a bit puzzling to see that the authors did not find evidence for increased model-based control in the L-tyrosine condition in the “model-agnostic” analyses. I expected the authors to address this point, but they did not. How is it possible that the effect is only found using the HDDM?

*Again, thanks for the opportunity to clarify this point. We did observe evidence for an increase in model-based control in the model-agnostic analysis of participants’ response times (see the results of the mixed effects regression reported in the manuscript). While participants showed faster response times in general following tyrosine, they showed a more pronounced slowing of response times in the second decision stage following a rare vs a common transition in the tyrosine condition. Importantly, the difference in S2 response times following rare vs. common transitions, as well as the model-based beta parameter of the DDM predicted reward rate throughout the task (see response to this Reviewer’s point 2 above). This demonstrates a direct link to task performance. However, the Reviewer is correct that in the model-agnostic analysis of S1 stay probabilities, we did not observe a significant effect. We apologize for the oversight of not having addressed this discrepancy in our manuscript. We now added a section in the discussion in which we elaborate on this (lines 831-43). This discrepancy may reflect that participants are more aware of the transition structure following tyrosine supplementation, as evident in the enhanced response time differences for rare vs. common transitions, and this effect is linked to task performance. However, the one-trial-back regression analysis might be less sensitive to pick up this effect, as compared to the DDM, which integrates across all trials.*

(5) It appears to be the case that the effective learning rates for the two-step task model can become higher than 1 or lower than 0 (given the parameters that indicate the L-tyrosine induced shift). This seems problematic. How do the authors justify this theoretically? How often did these extreme cases occur?

*We apologize for lack of clarity. The learning rates are fitted in standard normal space, meaning they can take on values from [-4,4] and are then back-transformed into the usual interval (0,1) via the cumulative density function of the standard normal distribution (sometimes referred to as ‘Phi’) within the model. We changed the respective lines in the methods modeling section to make this more explicit (lines 298-301).*

(6) I missed a discussion of Westbrook’s recent Science paper on dopamine and effort costs, which would nicely fit in with findings in both tasks.

*We thank the reviewer for this fruitful advice. We added a corresponding section in the discussion section of the manuscript (lines 821-25).*

(7) Also, I saw a lot of papers from Colzato’s former group, and I want to make the authors aware that this PI has been undergoing a lot of scrutiny for research misconduct. If they did not know about, I think it would be worthwhile to read up on this situation.

*We are aware of the accusation of research fraud under Prof. Colzato. We have not included any retracted paper of Colzato’s group.*

(8) Related to this latter point, there has recently been some very well justified criticism on the hypothesis that spontaneous EBR predicts dopamine availability. The authors can contribute to this burgeoning literature, but they do not cite one of its key players: <https://www.ncbi.nlm.nih.gov/pmc/articles/PMC5602106/>

We agree with the reviewer and added two papers (including the one proposed) into the *Methods* section (lines 163-64) to draw a more critical picture of eye blink rate as a proxy measure for central dopamine signaling.

Typos:

Equation on line 277 is wrong (unless everything right from Qmfs1 == 0), I think the left-most Q value should be indexed as t+1.

*We disagree with the reviewer here. This is the standard notation of a SARSA learner. The Q-value is updated at the end of a trial t in the light of encountered reward and state prediction errors in this trial.*

Line 303 misses closing parenthesis

*We corrected this mistake.*

Line 482: “proved to be a good test-retest reliability (Table1; Figure 1a)

*We corrected this mistake.*

Line 801-802: this sentence is not complete.

*We corrected this mistake.*

Reviewer #2

(1) There is no effect of tyrosine on model-based control in terms of choices, as is evident from the model-agnostic analysis of stage 1 stay probabilities and the softmax model results. However, tyrosine is shown to affect reaction times. Most notably, tyrosine increases RT slowing on stage 2 following a rare transition; this might reflect some surprise that stems from participants’ awareness of the task structure (i.e., a model) and its transition probabilities. However, despite this more pronounced surprise response to a rare transition, tyrosine did not affect the reliance on these transition probabilities when deciding between stage 1 options. To what extent, then, can we conclude that tyrosine enhances model-based control rather than tyrosine enhancing the acquisition / awareness of task transition probabilities? That is, tyrosine seems to facilitate the acquisition of a task model but does not seem to promote the reliance on that model in choice behavior.

*We thank the reviewer for raising this important concern. We agree with the reviewer that our findings may suggest that participants appeared more aware of the transition probabilities following tyrosine than following placebo but that there is no evidence in model-agnostic analysis of stay-switch behavior indicating that participants actually used this knowledge in their respective choices. The greater awareness of the transition structure may partly relate to enhanced working memory capacity following tyrosine (Thomas et al., 1999).*

*Nevertheless, we do see an increase in response time differences in stage S2 and in the model-based beta parameter in the DDM_s_ following tyrosine. Thus, differences in model-based Q-value estimates had a stronger impact on evidence accumulation under Tyrosine. More importantly, the difference in S2 response times following rare vs. common transitions, as well as the model-based parameter of the DDM showed a significant association with overall payout / reward rate (lines 571-73 & 595-98). This demonstrates that the RT effect is directly linked to performance, which argues against the idea that it only reflects awareness. The discrepancy between model-agnostic analysis of choice behavior in stage S1 (in terms of stay probabilities) and DDM analyses was also raised by Reviewer 1, point 4 above. We believe that this discrepancy is largely due to the reduced sensitivity of the one-trial-back regression model that is typically used for the analysis of stay probabilities. This is also supported by the observation that the model-based drift rate modulation parameter showed robust associations with performance (r>=.39) (lines 595-98).*

(2) The posterior predictive checks in figure 1 of the supplementary material suggests that the models predict a rather linear increase in RT across low to medium-high decision conflict, whereas participants’ RTs is clearly nonlinear. To what extent is this a failure of the models to capture a key signature in the data? Can the authors elaborate on this discrepancy and how this might affect the results / conclusions?

*Indeed the DDM seems to slightly overpredict the effects of a high decision conflict on participants’ response times in stage S1 of the seq. reinforcement learning task. However, the DDM_s_ shows a slightly better predictive performance than the DDM with a linear modulation of drift rates by value-differences (DDM_lin_). Importantly, this is only observable for response time patterns in stage S1 and here participants’ response times only marginally differ with respect to value differences. The DDM_lin_ and DDM_s_ are very well capable of predicting non-linear effects on response times as evident in the posterior predictive checks for response time patterns in stage S2. Here, participants response time differences regarding decison conflict are much more pronounced. We believe that it is unlikely that the slight overprediction of decision conflict modulation of response times in S1 have affected our main results. Rather, model-based signatures during reinforcement learning in this task might manifest more robustly in participants’ choice patterns in S1 and in response time patterns in S2. Both effects are well accounted for by the DDM_s_.*

**Reviewer #3**

 (1) Most importantly, I was surprised to read that the choice for the reinforcement learning model is not fully in line with previous realizations in the literature. While most modelling choices follow standard procedures, the stage1 choice rule (equation 8) deviates from previous instances of similar reinforcement learning models. To my knowledge, the conventional procedure to convert (and combine) model-free and model-based action values into action probabilities is (1) to derive net action values based on a model-basedness parameter and then (2) submit those net action values to the softmax decision rule. This is how it is reported in both early work (e.g. Gläscher et al. 2010) as well as a current DDM RL framework (Shahar et al., 2019). In contrast, the authors take a different approach. They refrain from calculating net action values based on a model-basedness parameter but rather introduce separate temperature parameters for model-free and model-based action vales within their softmax decision rule. Although I acknowledge that the softmax model is only used for a comparison purpose to the DDM models, a justification for the deviation from standard model formulations is missing in the current manuscript. Arguably, the deviation in model specification introduced by the authors has a significant impact on interpretability of the model. Moreover, it is not fully clear to me how the different parameters for the authors' model (separate model-free and model-based temperature) relate to the standard model (model-basedness and temperature). I would be interesting to learn if the two different model instances are recoverable.

*Thanks for the opportunity to clarify our modeling choices. Our modeling approach of the stage 1 choice rule is adopted from Otto et al. (2013). This choice rule formulation is algebraically equivalent to the more standard formulation of Daw et al. (2011) with the substitution* $\beta_{mb}=\omega\beta$ *and* $\beta_{mf}=(1-\omega)\beta$*. However, as Otto et al. (2013) discuss at length in the supplement of their paper, this reformulation facilitates group-level modeling of individual differences in the influence of either (model-free and model-based) system. Furthermore, in our experience, this (algebraically equivalent) reformulation leads to better convergence of the MCMC sampling procedure, as well as more efficient model estimation.*

 (2) I find the conclusion that model-based RL was improved by tyrosine (line 864) to be misleading. DDM shows that the beta parameter significantly improves for tyrosine. However, this scaling parameter (similar to the temperature parameter in the softmax model) is not involved in learning per se but only in decision making, i.e. translating learned values into action. Given the current form of the computational model, no statements about (the strength of) learning in a reinforcement learning framework can be made. For such a claim, a different model (e.g. model-free and model-based learning rates) or more event-related measures (e.g. EEG, fMRI or pupillometry) seem necessary.

*We thank the reviewer for this remark. With improved model-based reinforcement learning or model-based control (as we mainly call it in the paper), we refer to increased control of the model-based system (vs. the model-free system, in the sense of a dual-systems theory) that manifests in a stronger impact of model-based value computations on choice behavior during reinforcement learning. We rephrased the related section in the discussion to make this clearer.*

(3) Throughout the manuscript, the visualization could be improved. Personally, I believe visualization to be a key factor for promoting understanding of complex finding. As a positive example I have to highlight the use of violin plots which allow to depict information in an intuitive manner across both the individual as well as group level.

(3.1) The manuscript could profit from a figure that illustrates the design and structure of the tasks. Especially the sequential RL task is quite complicated. Understanding for readers who are not familiar with the task (or its modifications based on Kool et al.) would be fostered.

*We thank the reviewer for this advice. The revised manuscript now includes an illustration of the seq. reinforcement learning task (Figure 2 a, b).*

(3.2) On a similar note. In order to visualize the model-free and model-based contributions as revealed by the model-agnostic analysis, I suggest that the authors add a figure with both hypothetical (i.e. pure model-free and model-based) as well as empirical stay probabilities per condition.

*We added both suggested figures into the revised manuscript (Figure 2 c, d).*

*In addition, we also redesigned the figures that depict the posterior distributions of the DDM_s_ of both tasks to further enhance the visualization of the key findings (see Figure 5, 7 in the revised manuscript).*

(3.3) In order to highlight plot differences within and between figures, I prefer to use captions.

*This is obviously a matter of taste. We have decided to add the conditions (PLC / TYR) into respective figures to highlight their core content.*

(4) Contrary to my expectations, administration of L-Tyrosine seemingly leads to a decrease in physiological arousal, as indicated by pupil dilation and heart rate. Although the authors point to relevant literature on possible hypotensive effects of Tyrosine in stressful situation, the counterintuitive nature of the findings seems to be underrepresented in the current manuscript. Increased dopamine and noradrenaline plasma levels are reportedly associated with an overall (if mild) increase in physiological arousal.

*We understand the concern raised by the reviewer. Admittedly, one might expect increased physiological arousal following tyrosine administration due to potentially increased noradrenaline release. However, as we stated in the discussion section, there are animal and human studies, that link tyrosine supplementation to reduced stress levels, at least under high performance demands or stress. As we measured baseline physiological arousal directly after participants’ had entered the lab, participants presumably experienced somewhat increased arousal. This was also mirrored in a general reduction of physiological arousal in the second autonomic arousal assessment following placebo. And this reduction was more pronounced under tyrosine compared with placebo. We rephrased the discussion section (lines 735-38; 740-42; 745-46 & 749-54) accordingly to discuss this finding more critically.*

(5) Contrary to the data and code availability statement, modeling code is not available on OSF.

*We apologize for the inconvenience. All modeling code is now available on OSF.*

FURTHER COMMENTS

- Why are the variables LL magnitude and delay median-split before submission to the generalized mixed effects regression of the model-agnostic analysis of the temporal discounting task? Admittedly, I am not an expert for analyses of this task, but a median split seems to be overly simplistic, given the continuous nature of the two variables (16 and 8 steps respectively).

*Thanks for raising this point. We had initially run regression models with the raw delay and LL magnitude values, as suggested by this Reviewer. However, these mixed effects regression models failed to converge with the standard tolerance parameter set to .002 according to the lmer / glmer output. Notably, the results remained similar.*

- Although the methodology for the parameter comparison of the temporal discounting DDM is sound, the reported results need further clarification, as the sole strong effect (decision t*hreshold, see fig 6f) is not obvious in the main text (line 627-631).*

*We thank the reviewer for this remark. We now emphasize this consistent effect of reduced decision thresholds within the results section (lines 670-74).*

- Line 528: Figure 1b should read 2b

*We corrected this mistake.*
